# The PAX Good Behavior Game: One Model for Evolving a More Nurturing Society

**DOI:** 10.1007/s10567-020-00323-3

**Published:** 2020-08-25

**Authors:** Magnus Johansson, Anthony Biglan, Dennis Embry

**Affiliations:** 1 Department of Behavioural Science, Oslo Metropolitan University, St. Olavs plass, P.O. Box 4, NO-0130 Oslo, Norway; 2 Oregon Research Institute, Eugene, USA; 3 PAXIS Institute, Tucson, USA

**Keywords:** Nurturing environments, Evidence-based kernels, PAX good behavior game, Changing cultural practices

## Abstract

This paper describes the culture and components of the PAX Good Behavior Game and offers it as one model for how to enhance the well-being of populations through the diffusion of nurturing practices into several venues of society. The PAX components, also known as evidence-based kernels, are proposed to be useful in classrooms, families, organizations, criminal justice, and in improving public discussion and government. Kernels affect behavior in the short- and long-term through combinations of antecedents, reinforcers, relational networks, and physiological effects. Identifying common strategies, tools, and clear targets of change is suggested as a way to work towards evolving freely available evidence-based tools that can be combined to improve social conditions in multiple contexts.

## Introduction

This paper presents the PAX Good Behavior Game (PAX GBG/system; Embry, Fruth, Roepcke, & Richardson 2016) as one model for how society might evolve a more nurturing culture in a wide variety of settings beyond schools. We do not propose the implementation of the PAX system in other settings. Rather, we seek to use it to illustrate how a carefully organized set of interlocking behavior-influence kernels (Embry & Biglan 2008) could be relevant to promoting nurturance and prosociality throughout society.

One of the most persistent findings in prevention research is the fact that multiple problems result from stressful environments that fail to support the development of a variety of prosocial behaviors and values (Biglan et al. 2004; Dishion & Snyder 2016; Felitti 2009, 2017; Miller et al. 2011). Accordingly, most evidence-based interventions have components designed to reduce stressful social interactions and increase positive support for many forms of prosocial behavior, both of which are key aspects of nurturing environments (Biglan 2015; Biglan et al. 2012). Behavioral scientists are increasingly seeking ways to disseminate interventions widely and effectively (Brownson, Colditz, & Proctor 2017). From this perspective, it makes sense for practitioners, scientists, community leaders, and policymakers to explore diverse ways of promoting the spread of practices that nurture people’s well-being.

The PAX system grew out of efforts to strengthen social supports for the implementation of the Good Behavior Game. The Good Behavior Game (GBG) was originally developed by Barrish, Saunders, and Wolfe (1969). In that study, children in a classroom were divided into two teams, each of which could receive a reward of thirty minutes of free time at the end of the school day if the team had five or fewer instances of disruptive behavior while the game was being played. In an interrupted time-series design that alternatively evaluated the impact of the game on disruptive behavior first during mathematics instruction and then during reading instruction, the game was found to significantly reduce disruptive behavior. In the PAX system derivation of the original GBG, the term “PAX” stands for “peace, productivity, health, and happiness,” and PAX is the trademark of PAXIS Institute’s version of the Good Behavior Game.

**Fig. 1:**
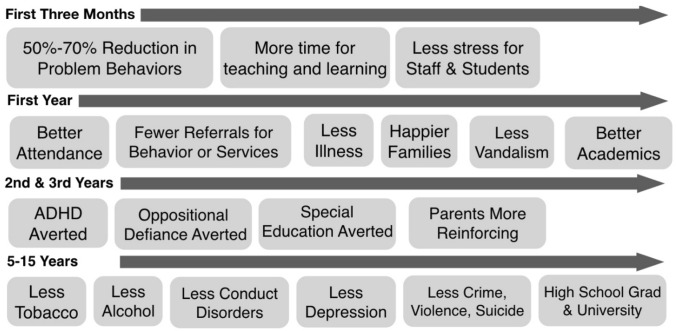
Impact of the Good Behavior Game in the Kellam studies

Since that original study, GBG was tested in a sizeable number of interrupted time-series designs (Embry 2002; Tingstrom et al. 2006; Flower et al. 2014; Bowman-Perrott et al. 2016). These studies generally showed that the game—even with different variations—reduced disruptive behavior in special education classrooms, as well as for special education students in regular classrooms and preschool students. However, because the behavior analysts who developed and tested the game in these studies were disinclined to conduct randomized controlled trials, it was not until the mid-1980s that GBG was evaluated in a randomized controlled trial. Sheppard Kellam, a psychiatrist at Johns Hopkins University, led a study in which children and teachers were randomized to classrooms, and the classrooms were then randomized to receive or not receive GBG. The manual, training, and coaching in the original Hopkins’ GBG study were created and delivered by Jaylan Turkkan (1985). It was substantially more sophisticated than the first studies on GBG reviewed by Embry (2002). Figure 1 summarizes longitudinal key findings from the first randomized, comparative effectiveness trial of GBG, versus Mastery Learning, and versus control in same schools (Dolan et al. 1993; Kellam et al. 2008, 2011; Kellam & Anthony 1998; Kellam, Ling, Merisca, Brown, & Ialongo 1998a, b; Kellam, Rebok, Ialongo, & Mayer 1994). Notably, the GBG is one of the few universal prevention interventions that has shown an effect on reducing suicide attempts (Wilcox et al. 2008) by increasing peer reinforcement for prosocial behaviors while also decreasing problematic behaviors that harm positive peer relationships (Newcomer et al. 2016).

## The Culture and Components of PAX Good Behavior Game

In the PAX Game (the GBG itself), teams of three or four students work on any academic activity, such as silent reading or solving math problems. The students play the Game for as little as three minutes initially, and as students get better at staying on task, the teacher gradually increases the length of the game. In the PAX Game, teams that have three or fewer disruptive behaviors participate in a joyful voluntary activity as a reward, immediately after finishing the Game. Based on the Premack principle (Klatt & Morris 2001), the rewards are typically things that students aren’t allowed to do in class, rather than tokens or tangible rewards.

However, before the game is introduced, teachers implement a set of practices that facilitate the implementation of the game and create a culture that supports self-regulation and cooperation. These practices are called *evidence-based kernels* (Embry & Biglan 2008). They are simple behavior-influence techniques, which in experimental research have shown a consistent impact on a specific set of behaviors. Embry (2011) described kernels as

an indivisible procedure empirically shown to produce reliable effects on behavior, including psychological processes. The unit is indivisible in the sense that it would be ineffective upon elimination of any of its components. Examples of kernels include timeout, written praise notes, self-monitoring, [and] framing relations among stimuli to affect the value of a given stimulus [...]. A kernel may increase the frequency of a behavior or it may make a behavior less likely. It can have its impact by altering antecedent or consequent events in the psychological environment of the person or it can affect behavior by directly manipulating a physiological function. (p.10)

Repeated use of kernels throughout the school day provides students with many opportunities to practice prosocial skills and self-regulation. Embry (2011) coined the term “behavioral vaccines,” based on how a combination of kernels used repeatedly can lead to increased well-being and lowered risk for detrimental long-term outcomes. The addition of kernels to the GBG is the most salient way that PAX GBG differs from other versions of GBG.

Table 1 presents a list of the kernels used in the PAX system. We indicate whether they are focused primarily on the modification of relational networks, the reinforcement of behavior, or the antecedents to desired behavior. Kernel names are written in italics throughout the text.

**Table 1:** Kernels included in the PAX system

	PAX component	Type of evidence- based kernel
1	PAX Vision	Relational network
2	PAX Leader	Relational network
3	Predict, Monitor, Reflect	Relational network
4	Granny’s Wacky prizes	Reinforcement/ Physiological
5	Tootle Notes	Reinforcement
6	OK/Not OK	Reinforcement/ Relational network
7	PAX Sticks	Antecedent
8	PAX Quiet	Antecedent/ Physiological
9	Beat the Timer	Antecedent

## Cultivating Relational Networks that Support Prosocial Behavior

Prior to any implementation of the game itself, teachers introduce a set of kernels, each of which is intended to contribute to a *shared relational network*. Research on the ability of humans to arbitrarily relate stimuli has shown that the majority of effective consequences for verbally able humans result from the transformation of stimuli via their relation to other stimuli (Dougher & Hamilton 2018; Dougher, Hamilton, Fink & Harrington 2007). Relational frame theory (RFT; Dymond & Roche 2013; Hayes et al. 2001) provides the foundation for this research, although there is very little experimental research directly related to RFT and relational networks in school settings. As applied to schools, RFT implies that the network of verbal relations that children and teachers have about themselves, their peers, and behavior affects their behavior and the behavior of those around them. Students and teachers may have a set of relational networks that support prosocial behavior or they may have networks that undermine such behavior and promote coercive behavior.

For example, in the PATHS program (Greenberg et al. 1995), children learn about emotions. Consider “sadness.” Students are taught what kind of facial configurations, express sadness, what it feels like to be sad, what kinds of things make kids feel sad, things that you can do when you feel sad, and how others act when they are sad. Taken together, all of these relations can be thought of as a relational network about sadness, and all of the networks about all the emotions can be thought of as the child’s relational network about emotions. When children in a classroom participate in learning about these same relations, the class might be thought to have a shared relational network about emotions. Having a shared understanding of emotions likely facilitates social relations by promoting understanding and empathy about others’ emotions.

One of the key facets of a culture is the degree to which the members of a group shared the same beliefs, attitudes, and norms about behavior. For example, in some places, a gun culture consists of networks of people who enjoy owning and using guns, share a set of beliefs about the importance of owning guns, fear that guns will be confiscated, etc. (Metzl 2019).

We submit that in most elementary school classrooms, children’s relational networks about appropriate social behavior are limited to an understanding of a set of rules that the teacher has established and their experience in seeing what happens when rules are broken or prosocial behavior is recognized, rewarded, or ignored. The PAX system was evolved to strengthen children’s—and teacher’s—relational networks about contextually appropriate and inappropriate social behavior.

From this perspective, an essential process in establishing students’ successful behavior is to build a network of verbal relations that increase the positive valuing of prosocial behavior, thus making it more reinforcing to engage in such behavior. RFT does not replace the core learning principles of antecedents, positive reinforcement, and reduction of reinforcement of problematic behaviors that have been foundational in educational settings, it helps leverage those principles for generalization across people, places, activities, and time.

### PAX Vision

On the basis of this theoretical analysis, the PAX system employs a set of kernels that cultivate shared relational networks that identify and promote specific forms of prosocial behavior and discourage behavior that is inconsistent with prosociality. The opening gambit in the process of implementing the PAX system is jointly creating the *PAX Vision*. The teacher asks students what they would see, hear, feel, and do, “if this were the most wonderful classroom you could imagine.” Then, the teacher asks them what they would see, hear, feel, and do *less of,* “if this was the most wonderful classroom.” Their answers go onto a large poster, which remains prominently posted in the classroom throughout the year, often referred to, and updated when necessary. While the Vision process can seem similar to standard procedures to create classroom rules, it is a highly participatory process, where the students play a central role in creating the vision, which is different from a top-down process where the teacher establishes the rules or values and, at best, might ask the students for examples. Participatory processes increase commitment to what is specified (Mager & Nowak 2012) and reduce the tendency for oppositional responses from students. This group process results in students developing a *shared* set of norms, which recruits the social influence of other students in support of the specified norms.

The teacher labels the things students would like to have more of as “PAX” and the things students would like to have less of are labeled “Spleems,” a made-up word with no prior relational network attached to it that is intended to refer to undesirable behavior in a neutral, non-emotional way, aiming to minimize stressful and stressing reactions to undesired behavior and, instead, promote a calm, collegial approach to having fewer Spleems. The neologism was explicitly designed to reduce previously conditioned autonomic arousal from words like “bad” or “negative”.

It is important to understand the way in which the word “PAX” functions in the relational network the teachers and students are establishing. It stands in a hierarchical relation to a whole network of concepts and behaviors (Gil-Luciano et al. 2017), meaning that the value of a given stimulus (e.g., a word referring to a behavior) is affected by the stimuli the child relates to that stimulus. By using the word, group members can invoke not simply one concept, but the entire shared vision of the culture of the classroom or school that was established by relating PAX to a variety of positively valued words and behaviors. Second, by labeling a behavior as PAX, one can immediately enhance the value of that behavior for the listener due to its newly created relationship to the existing network of valued words and behaviors. Using the *PAX Vision* operationalizes the original Turkkan (1985) GBG manual that said: “…consider letting students participate in defining target behaviors and setting criteria. There is some evidence that if children are allowed to help in setting their own standards and contingent tasks, the probability of on-task behavior increases” (p. 6).

Teachers and students continue to use *PAX Vision* throughout the year, often on a daily basis, to build norms for prosocial behavior, and to expand the network of behaviors related to PAX and those related to “Spleems.” In this way, *PAX Vision* provides the initial framework for making a broad array of prosocial behaviors more reinforcing. In general, most kernels involve verbal descriptions of PAX or Spleems, which contributes to the development of the shared network of relations that motivate PAX and discourage Spleems. The *PAX Vision* functions as an overarching framework, connecting all PAX kernels to the students’ shared norms.

### PAX Leader

The *PAX Leader* kernel engages students in self-modeling (Hitchcock et al. 2003) in which they describe things that they and other students do to promote PAX. Each time students talk about what they or others do to create PAX, they are elaborating their relational network about PAX. Students also nominate other students as *PAX Leaders*, thereby providing social reinforcement for diverse forms of prosocial behavior. Meaningful roles give students things to do in the classroom or school that communicate trust in the student. These roles further expand the ways in which prosocial behavior becomes reinforcing. Each role involves prosocial behavior. Examples include taking the roll, organizing bulletin board displays, and greeting fellow students as they come into class. Such positive roles elicit peer reinforcement for prosocial behaviors (Ellis et al. 2016).

In addition to drawing on research on the impact of relational networks on motivation (Hughes & Barnes-Holmes 2016), kernels that expand relational networks about PAX are consistent with research in social psychology that shows the value of participation in decision-making for motivating behavior (Zimmerman & Rappaport 1988) and on evidence that humans are more likely to engage in specific behaviors when they associate those behaviors with important values (Cialdini & Goldstein 2004; Deci & Ryan 2008). This is also congruent with the vast research on self-determination theory (Ryan & Deci 2008) that emphasizes autonomy and relatedness as key factors to facilitate motivation.

## Increasing the Frequency of Reinforcement for Prosocial Behavior

The importance of reinforcing consequences for promoting prosocial behavior is one of the most established principles in behavioral science (Biglan, 2015). However, it remains a significant challenge to create systems in schools to increase positive consequences for prosocial behavior and to provide non-traumatic and effective consequences for reducing undesirable behavior. The PAX system provides six kernels that promote effective use of consequences.

### Granny’s Wacky Prizes

This kernel consists of a set of brief, simple, fun activities that students can engage in as rewards for playing the game successfully or engaging in other desirable behavior. Examples of *Granny’s Wacky prizes* include making animal noises for 30 s, arm wrestling, playing hangman, crumbling pieces of paper and throwing them around the room, and having a conga line. None of these rewards involves food or costs any money. Consistent with the Premack Principle (Premack 1962), most are activities typically prohibited in classrooms.

The selection and inclusion of the prizes bend toward physically active prizes for a variety of scientific and child-development reasons: (1) self-regulation is contextual, and the variety of actions, emotions, timing, numbers of people scaffold complex self-regulation skills, and (2) the physical activities have other health benefits based on medical research in better academics and physical health (Donnelly et al. 2009, 2013).

### Tootle Notes

*Tootle Notes* are praise notes. Skinner, Cashwell, and Skinner (2000) developed tootling, a peer-based class-wide intervention in which students reported on other peers’ positive, prosocial behaviors. They based the name of the intervention on a positive variation of tattling that often occurs in the classroom and is a derivation of the idiom, “tooting your own horn” (Lambert et al. 2015; Wright 2019).

With *Tootle Notes*, students as early as first grade learn to write praise notes to classmates. A 10-min exercise during which each student writes a praise note to another, often randomly chosen, student can infuse a classroom with recognition and appreciation for classmates. Teachers can also write *Tootle Notes* and children can write *Tootle Notes* to teachers. Children are encouraged to bring notes they receive home. This can prompt parents to provide further positive support for their child’s behavior. *Tootle Notes* massively increase the level of recognition, praise, appreciation, which improves prosocial skills and interactions with peers, particularly for socially withdrawn students (Nelson et al. 2008). Moreover, *Tootle Notes* further expand children’s relational networks about PAX by associating all of the praised behavior with PAX.

### Small Cards with the Words “OK” and “Not Ok”

The *OK/Not OK cards* go on student’s desks and the teachers’ lanyard and enable the teacher simply to touch OK or Not OK to signal a mild, non-verbal/-auditory consequence for behavior. The cards’ design minimizes student arousal and avoids debates about their behavior. The PAX system encourages teachers to provide at least five OKs for every Not OK. The aim of the *OK/Not OK cards* is also to reduce teacher verbal reprimands (Caldarella et al. 2020), which worsen children’s behavior.

## Antecedents to Prosocial Behavior

The PAX system also makes use of three kernels whose primary function is to prompt desired behavior.

### PAX Stix

There is evidence that using random calling, which the PAX system labels as *PAX Stix,* with students increases engagement and learning (Keen 2006; Maheady et al. 2002; Martino & Sala 1996). The teacher prepares a container with popsicle sticks, with each stick having the name of a student in the class. It functions as an antecedent to paying attention to the teacher’s behavior because students know that the teacher may call on them. *PAX Stix* also scaffolds the ability of shy, anxious, or socially isolated children to participate.

### Beat the Timer

The *Beat the Timer* kernel challenges students to complete a task efficiently. For example, after establishing a baseline, the teacher may challenge students to make the transition from one activity to another in less than two minutes. *Beat the Timer*, which is called “reduced allocated time” in the programming of most electronic games, is a key factor in holding attention. School staff learns to provide *Granny’s Wacky Prizes* often for beating the timer efficiently and with proper behavioral control as a group.

### PAX Quiet

*PAX Quiet* is designed to get every students’ attention in a way that is not harsh or stressful for students who may be anxious. The teacher blows on a harmonica and makes the peace sign. Children are taught to pause what they are doing, stop talking, make the peace sign, and look at the teacher’s face as an indication that they are paying attention. This kernel facilitates much faster transitions between activities which are important because transitions can consume considerable amounts of time every school day. According to a recent poll (EAB 2019), American teachers estimate that on average they lose 144 min of instruction time each school week due to disruptive behavior. *PAX Quiet* also reduces harsh interactions from adults trying to manage transitions.

### Kernels That Affect Physiological Functioning

Brief vigorous physical activity increases alertness and concentration. Sibley and Etnier (2003) found “a significant positive relationship between physical activity and cognitive functioning in children.” Of course, it also promotes health (Perry et al. 1997). Opportunities for such activity is highly reinforcing in the context of a classroom, which mostly requires inactivity. Many of the *Granny’s Wacky Prizes* involves physical activity. These prizes thus serve the dual functioning of reinforcing behavior and prompting healthy physical activity.

### The Interaction Among PAX Components

*Predict, Monitor, and Reflect* are designed to integrate kernels involving relational frames, antecedents, and consequences. Before initiating a classroom activity, the teacher asks students to describe some of the PAX and Spleems named in their *PAX Vision* that are relevant to the activity. During the activity, teachers look for examples of PAX that students have suggested and provide verbal or non-verbal social reinforcement. At the end of the activity, the group has the opportunity to report if they have observed any of the items from their vision during the meeting and they receive encouragement to provide examples. This activity increases reinforcement of PAX and gives friendly reminders about Spleems.

Virtually all components of the PAX system have multiple functions. Developing a shared vocabulary about desirable and undesirable behavior influences behavior through the principles involved in rule-governed behavior (Hayes et al. 1986). That is, most human behavior is a function of verbal relations. However, without positive consequences that agree with verbal rules, rule-following is unlikely to endure. In PAX, there impli

s a continuous interplay between cultivating relational networks and providing positive consequences for behavior that corresponds to children’s relational networks. Similarly, if a teacher awards a *Granny’s Wacky Prize* when all students respond quickly to *PAX Quiet*, it strengthens children’s response to the teacher’s signal.

## The Critical Role of Self-Regulation

Children’s development of self-regulation is critical to their academic and social success (Tangney et al. 2004). PAX components provide daily experiences that are likely to strengthen self-regulation (Mulgrew 2019). For example, every time children engage in a high-rate, exciting activity during *Granny’s Wacky Prize* and afterward *PAX Quiet* brings them back to focus on work, they are getting better at inhibitory control. Each time students stay on task during the game, they are practicing self-regulation. If a team fails to get a prize and has to sit out involvement in a *Granny’s Wacky Prize*, it provides an opportunity to practice emotional regulation. When a teacher touches “Not OK” and the student does not get upset, it is an example of emotional regulation. When a teacher asks a question and uses *PAX Stix* to select the respondent randomly, those who usually seek attention need to regulate their responses down, while those who might usually avoid participation increase their activity level. When a student team does not succeed at a *PAX Game*, that helps students practice self-regulation when disappointed.

When the kernels have been established in the classroom, the *PAX Game* is introduced. The length of games is initially only a few minutes, but as students’ ability to work cooperatively in small groups grows, the length is extended. The most important thing about the game is that it involves students working cooperatively together to succeed in the challenge presented by the *PAX Game*. What gets selected in this way is the interlocking behavior of the group (Glenn 2004; Malott & Glenn 2006), and co-regulation of individual behavior within groups. The *PAX Game* helps to strengthen the behavioral inhibition part of self-regulatory skills.

## The Benefits of a System Approach

One might argue that the PAX system is merely a collection of classroom management techniques. However, deconstructing it in this way overlooks at least two things. First, each kernel strengthens shared relational networks and increases the likelihood of prosocial behavior. Taken together, the kernels not only extend each student’s relational network about PAX but also establish a shared understanding of PAX among students that increases the chances that students will respond positively to each other’s PAX behavior. Second, the implementation of the kernels is supported by a well-developed training system and a set of materials that support implementation, which teachers receive during the initial training.

## The PAX Delivery Method

The training of teachers utilizes all of the PAX system kernels during training, to maximize participatory and experiential learning. The PAX trainers work to make the training become a nurturing environment for the teachers, by creating a *PAX Vision* for the training, and working with the teachers in a similar fashion to how they will work with their students. Theory and lecturing are kept to a minimum; focus is on learning how to use the kernels. While PAX trainers model use of the kernels, teachers also practice introducing and using each kernel in small groups. All materials needed for PAX in the classroom is provided during training, to avoid practical obstacles when beginning implementation. The materials include a PAX handbook, which provides concrete suggestions for how to introduce every component, things that are extra important to do and not do, and suggestions for troubleshooting if needed. The implementation roll-out is scheduled by teachers at the end of training, based on a suggested order of introducing the PAX components, so that they leave the training with an implementation plan already in place. The order of components is based on the strategy of “early wins,” ensuring that the teacher will experience natural reinforcement immediately when introducing the first kernels, scaffolding the motivation for implementing more complex kernels later on. *PAX Quiet* in combination with *Granny’s Wacky Prizes* is a good example of an “early win.” The teacher instantly receives students’ attention without raising their voice, and the students and teacher enjoy the *Prize* intermittently, used to reinforce attentive behavior.

Teacher training typically takes 1–2 days, sometimes combined with a later booster session. As implementation moves along, teachers fill out their roll-out schedule. They are also provided with a checklist for self-monitoring the use of different components, which matches the checklist used by trainers at supervision visits. The initial training session is usually followed by at least 2–4 classroom visits by a trainer who observes and supervises each teacher individually, based on their needs (Becker et al. 2013a, b). On-site coaching with observations and performance feedback has been found to be effective implementation strategies (Becker et al. 2013a, b; Fallon & Kurtz 2018; Joyce & Showers 2002; Powell et al. 2015; Reinke et al. 2014; Stormont et al. 2015). Self-monitoring with checklists can also be helpful (Copeland et al. 2018; Olson & Winchester 2008; Webster-Stratton et al. 2011), not least for maintaining fidelity over time (Oliver et al. 2015).

## A Brief Review of the Evidence Regarding the PAX Good Behavior Game in Schools

Building on the important work of Kellam and his colleagues, which established the long-term benefits of the GBG (Kellam et al. 2011, 2014; Kellam et al. 1998a, b; Poduska et al. 2008), the PAX GBG has been further developed by Embry and colleagues (Embry et al. 2016). Successively more elaborate versions of this system have been evaluated in a series of studies, including randomized trials and quasi-experimental designs.

As PAX GBG is a universal prevention intervention, the overall effects are likely to be small in terms of clinical effect sizes such as Cohen’s *d* (Greenberg & Abenavoli 2017) and provide greater benefits to risk groups, such as documented in the Hopkins’ studies. This is also consistent with genetic studies on “differential susceptibility” (Albert et al. 2015; Belsky & van IJzendoorn 2017; Boyce 2016).

### Randomized Controlled Trials

In an RCT conducted in the province of Manitoba, Canada, children in first grade in 197 schools were randomly assigned to receive PAX GBG in the 2011–2012 school year or to be in a waitlist control condition that received PAX in the subsequent school year (Jiang et al. 2018). Notably, the province-wide prevention strategy funding the study did not include coaching because of equity policy issues. Following up at the end of the school year, students in the PAX classrooms scored significantly better on teacher ratings of the Strengths and Difficulties Questionnaire (SDQ; Goodman 2001) compared with students in the waitlist condition, for all five SDQ subscales; Prosocial Behavior, Emotional Symptoms, Conduct Problems, Hyperactivity, and Peer Relationship Problems (Cohen’s *d* ranging from 0.11 to 0.23). Latent transition analysis indicated that children in the moderate- and high-risk categories on the SDQ were significantly more likely to transition to a lower risk category if they were in a PAX classroom (35.1% probability for medium risk and 44.7% probability for high-risk group, Jiang et al., 2018). Although sensitivity analyses were conducted and found no bias, the large attrition of SDQ-data both at pre- (30.7%) and post-intervention (37.9%) is a weakness in this study. Only relying on ratings by teachers, who are liable to bias as they are also delivering the intervention, is also notable. Other, more objective outcome measures would have added significantly to the strength of this study. Latent transition analysis is a suitable statistical model to analyze and report outcomes of a universal prevention trial beyond traditional effect sizes since most students do not have difficulties and are therefore unlikely to show significant improvements. Fidelity was determined by asking teachers at the end of the school year to fill out an implementation form on the extent of their PAX usage, which can be affected by recency bias and misestimation. The implementation form had an attrition rate of 50%, which undermines the possibility to draw conclusions.

In a second RCT, conducted in Baltimore, Ialongo et al. (2019) compared the impact of PAX with a “standard setting control condition," and a condition that integrated PAX with the Promoting Alternative THinking Strategies program (PATHS; Greenberg et al. 1995). The PATHS program teaches children about their emotions and social skills relevant to emotional regulation. At the measurement conducted 6 months after beginning the intervention, PAX combined with PATHS (PATHS to PAX) showed a small effect (Cohen’s *d* = 0.08) on independent observations of off-task, disruptive and/or aggressive behavior in the classroom. By using the Johnson-Neyman technique to identify regions of significance, the study found that students who were elevated in Total Problem Behavior at pre-intervention measurement had improvement on this outcome if they received PAX alone (Cohen’s *d* = 0.05). Similarly, the PATHS to PAX intervention also produced significant minor improvements on four teacher-rated variables, but only for those rated low on these variables at pretest: Readiness to Learn, Authority Acceptance, Social Competence and Emotion Regulation (Cohen’s *d* ranging from 0.03 to 0.09), measured with the TOCA-R (Werthamer-Larsson et al. 1991) and Social Health Profile (Conduct Problems Prevention Research Group 2010). However, there were no significant main effects for the PAX only (without PATHS) condition compared with control schools. No significant difference was found between the two PAX conditions regarding the dose of GBG use during the school year, nor in the fidelity ratings.

In a third RCT, conducted in Estonia (Streimann et al. 2017, 2015, 2019), PAX GBG was implemented in an initial ten first-grade classrooms to test its adaptation to the Estonian culture. It was then provided to 23 schools in a cluster-randomized trial with 23 schools allocated to a waitlist control. At the two-year follow-up, children receiving PAX had improved their overall mental health (Cohen’s *d* = 0.39) and reduced conduct problems, peer problems, and hyperactivity (Cohen’s *d* ranging from 0.17 to 0.24) on the teacher-rated SDQ (Streimann et al. 2019). No improvement was found for SDQ ratings of prosocial behavior. Subgroup analyses were done by using cut-off scores for the SDQ and calculating Odd’s ratios, but no statistically significant differences were found. The two-year follow-up period is a strength, this study would have been even stronger if it had reported independent data and not only teacher ratings as the primary outcome. Fidelity assessment in this study was exemplary, combining mentor ratings and ratings by independent researchers. The dose measure was less optimal, using a retrospective estimate by teachers at the end of the school year rather than collecting data on the number of games and their duration throughout the school year.

A pilot cluster-randomized controlled trial was conducted in Northern Ireland (O’Keeffe et al. 2017) with 353 children, ages 6–8, at 15 schools (19 classrooms) in areas of high socio-economic disadvantage. Short-term outcomes (12 weeks) reported in a thesis by Mulgrew (2019) indicate significantly improved self-rated student self-regulation (Cohen’s *d* = 0.42) compared to a passive control group. No other outcomes (SDQ and TOCA) were statistically significant after controlling for clustering on school level. As the study was a pilot trial, it suffered from low statistical power that was further affected by attrition, ending up with almost twice as large sample size in the intervention group compared to control at follow-up.

One RCT studied the use of PAX GBG in an afterschool setting with children in grades 2–5 (Smith et al. 2018), at 76 afterschool sites in diverse geographical areas. Sites were matched pairwise on relevant variables and randomized to either received PAX GBG or “business-as-usual.” Independent, blind to condition observers conducted observations on-site at two pre- and two post-intervention occasions. Fidelity was also independently rated. PAX GBG sites were found to improve observed belonging (γ = 0.23, *p* < 0.05), and child self-reported hyperactivity using the SDQ (γ = 0.76, *p* < 0.05), compared to control sites. Sites with high fidelity ratings also showed statistically significant effects on observer ratings of harshness/criticism (γ = -1.11, *p* < 0.05), supportive relations with adults (γ = 1.83, *p* < 0.01), appropriate structure (γ = 1.54, *p* < 0.01), and levels of engagement (γ = 1.82, *p* < 0.01). No subgroup analyses were conducted. The implementation procedure and outcomes, while outside the scope of this review section, were also studied in greater detail and published separately (Smith et al. 2014).

Summing up the RCT review, there is a fair amount of variation in outcome measures, statistical methods, and follow-up timespans in these studies. Several rely largely on teacher-reported evaluations of students’ progress, such as the SDQ and TOCA. While this is important data, the teachers are not independent raters (Pas & Bradshaw 2014). Increased use of independent behavior observations and objective data records, such as attendance and test scores, would strengthen study designs. The time span of follow-up varies from three months to two years and the Estonian study (Streimann et al. 2019) is the only one reporting outcomes at multiple time points (one and two years), which is helpful in understanding the development over time. There were three different approaches to exploring subgroup effects based on baseline measurements, Latent Transition Analysis (LTA), the Johnson-Neyman technique, and cut-off scores. Both Johnson-Neyman and cut-off scores use rating scale sum scores, which can be problematic (McNeish & Wolf 2020), while LTA also takes the measurement model and measurement error into account. LTA is likely to be the most robust analysis method, but it requires large datasets to be appropriate. All studies report Cronbach’s alpha for their measures, but they do so for pre- and post-measurement together for all groups. This may be described as standard practice, but it does not allow any insight into possible issues with measurement invariance between timepoints and groups. Most studies assessed both fidelity and dose delivered, which is praiseworthy, not least from an implementation perspective. However, the assessment methods could be stronger and more systematic. Retrospective ratings over long time-periods are likely to be unreliable. Finally, two of the studies published study protocols prior to conducting their trial, which is a commendable practice (Streimann et al. 2017; O’Keeffe et al. 2017).

## Quasi-experimental Trials

Four additional studies have been conducted in the context of disseminating the PAX system. Although these studies employed quasi-experimental designs, they can be viewed as useful for verifying that the system was having expected effects prior to a more extensive dissemination in a locality.

In Ireland, a pilot trial was conducted (O’Donnell et al. 2016), implementing PAX GBG in 21 first or second grade in schools that were designated as socially disadvantaged. A total of 420 students were involved. Evaluation after 12 weeks of using PAX, independently observed disruptive behavior on classroom level decreased significantly (Hedge’s *g* = 0.57). Significant improvements were also shown on teacher ratings of children on the SDQ total problem scale (Cohen’s *d* = 0.21) and prosocial behavior (Cohen’s *d* = 0.12). In addition, the percent of children who were categorized as “borderline or challenging” on the SDQ declined by 29%.

In Sweden, PAX GBG was culturally adapted, then pilot tested in 14 grade 1–2 classrooms in six schools (Ghaderi et al. 2017) with no control group. After five months of implementation, there were significant reductions in independent observations of children’s off-task and disruptive behavior on classroom level (Hedge’s *g* = 1.19), and significant improvements on teachers’ ratings on all subscales of the SDQ (Cohen’s *d* = 0.56 for Total problems and *d* = 0.82 for Prosocial behavior). Teachers’ perceived stress also decreased significantly (Hedge’s *g* = 1.70).

In Oregon, PAX GBG was introduced into three rural elementary schools over a two-year period (Biglan et al. 2017). In the first year of implementation, the rates of directly observed disruptive behavior declined significantly between the fall and the spring assessments (Hedge’s *g* = 1.14). During the same time period, teacher ratings of students on the SDQ improved significantly on the hyperactivity subscale (Cohen’s *d* = 0.22).

In Ohio, a quasi-experimental comparison of students in first through third grade in PAX vs. non-PAX schools (Weis et al. 2015) found that children in schools implementing PAX had greater improvements in math and reading achievement than students in the comparison schools. The effects were larger for boys with low pre-intervention scores. In a review of programs to improve mathematics in elementary school (Pellegrini et al. 2018), PAX GBG was found to be better than many math curricula.

While most of these quasi-experimental studies lack experimental control and rigor, several of them use strong measures such as direct observations of behavior and test scores, combined with teacher ratings. As previously noted, these studies can perhaps best serve as a reference for which short-term outcomes can be expected for those interested in evaluating their local implementation of PAX. The use of similar measures in many of the studies helps with the comparability of outcomes.

It should be noted that, as far as we know, there is no published experimental evaluation separating the potentially additive effects of PAX kernels to the Good Behavior Game itself. Nor are there any studies comparing PAX GBG to other versions of GBG. A quasi-experimental assessment of PAX kernels’ impact on the disruptive behavior in 186 classrooms in eight school districts indicated that their implementation significantly reduced the rates of disruptive behavior (Wilson et al. 2014, p. 408), before introducing the *PAX Game*.

## Implications of PAX for Other Settings

Hopefully, we have presented sufficient evidence to make it plausible that other venues of society could benefit from using a system of kernels to create a shared relational network that supports prosociality and nurturance. In the following sections, we describe how a similar network of kernels could help build strong prosocial cultures in families, workplaces, and juvenile offender institutions, as well as the quality of systems of governance.

## Families

There is strong evidence of the efficacy of family interventions focused on strengthening the quality of parenting (Leslie et al. 2016; Van Ryzin et al. 2016). However, we believe that many of the kernels that have proven useful in classrooms could strengthen family interventions. To engage families at a *population level* versus clinical practice requires high acceptability, ease of use, low-cost in terms access and time, early wins that inspire tackling harder issues, and public perception of positive status for adoption and use rather than designation as “at-risk” or deficient.

### Cultivating Relational Networks that Support Prosocial Behavior

Our review of a number of evidence-based parenting interventions led us to conclude that most of these programs are not doing as much as they could to help parents and children develop a rich relational network about the values and the behaviors that the family wants to promote (Lee et al. 2018). One indication of this is the meta-analysis of family interventions conducted by Van Ryzin and colleagues (2016), which found a significant benefit of a component in a small number of family interventions that promoted children’s positive orientation toward the future. In our view, the component was affecting children’s relational network about the relation between current behavior and its contribution to a positive future.

We believe that the process of parents leading their children in a *PAX Family Vision* exercise would have multiple benefits. By asking the children to say what they would like to see, hear, feel, and do more (PAX) and less (Spleems) of in their family life, the children participate in creating the goals for the family together with their parents. We believe that this joint participation increases children’s and parents’ commitment to the goals and values that they discuss. The terms PAX and Spleems could be exchanged for any preferred words, although we recommend using neologisms to avoid connections to previously learned relational networks, such as that bad behavior implies that you are a bad person.

Ongoing use of the vision the family creates should enrich children’s and parents’ relational networks in ways that would focus attention on prosocial behavior and reduce inadvertently reinforcing attention to problem behavior. For example, using *Predict, Monitor, and Reflect,* parents could briefly discuss an upcoming event with the child/children, such as a visit to the grocery store, in terms of PAX they want to see and Spleems they might avoid. Then, the parent can praise examples of PAX during the activity and can discuss together how they did in terms of PAX and Spleems at the end of the activity. Some evidence-based family interventions already do this to an extent. For example, Triple P teaches parents to discuss what will be appropriate and inappropriate behavior just prior to an activity such as going into a store (Sanders et al. 2012; Turner & Sanders 2011).

### Increasing the Frequency of Reinforcement for Prosocial Behavior

Increased reinforcement is already a key component of most evidence-based family interventions. *Granny’s Wacky Prizes* and *Tootle Notes* are concrete ways to increase parents’ reinforcement of PAX behaviors. The prizes provide the parents with brief reinforcing activities to replace or supplement any organized reinforcement system. They can enrich the variety of available rewards and avoid using food or costly rewards. Because they are brief, they are also efficient. Involving the children in creating a repository of prizes they like is likely to increase reinforcement.

*Tootle Notes* are another concrete way to help parents organize routine praise for children. For example, a parent might establish a routine time to write a *Tootle* to each child each week. Parents could prompt children to write *Tootles* to each other or other members of the family. The writing of *Tootles* also further elaborates children’s and parents’ shared relational networks about PAX. A fun family activity could be to use *PAX Stix* to identify a family member to write a note to.

### Antecedents to Prosocial Behavior

Getting children’s attention is the first step in most sequences of interactions—asking children to help set the table or asking them to be quiet. Given the variety of situations in which parents need to get their children’s attention, using a harmonica, such as teachers do in *PAX Quiet* may not be practical. Instead, parents can establish a pleasant visual or verbal stimulus: e.g., the peace sign or a made-up word said in a warm way. The child might participate in making up the word. Key to the effectiveness of any antecedent is the reinforcement of a positive response to it. A parent could establish this sequence by practicing the antecedent-behavior response and expressing gratitude or on some occasions following it with a *Granny’s Wacky Prize*.

*OK and Not OK* signs can be particularly valuable to parents in cars, while shopping, in church, or at public events as well as at home. It reduces emotional responses by parents when frustrated with a child.

Because many of *Granny’s Wacky Prizes* involve high-rate and typically prohibited activities, they also provide opportunities for children to practice self-regulation. After establishing an antecedent signal through practice and rewards, using it at the end of a prize provides a good opportunity to practice self-regulation.

*PAX Stix* (random calling) may also be helpful in families. In activities involving multiple children, it will ensure equal amounts of attention and increase children’s sense of fairness. The whole family can be involved with *PAX Stix* so that the parents also take turns in various activities.

### Kernels That Affect Physiological Functioning

Many of the *Granny’s Wacky Prizes* involve high levels of physical activity for brief times. Such interludes of activity may help to increase children’s concentration (Hillman et al. 2011; Mahar et al. 2006). Moreover, many children are less active than is good for their health. According to a nationwide study, only 42% of children ages 6 to 11 and 8% of adolescents ages 12 to 19 achieve the recommended 60 min of physical activity per day (Troiano et al. 2008). Parents might collaborate with their children in developing *Granny’s Wacky Prizes* that involve more extended or intense physical activities they can engage in together. Increasing the physical activity of children is associated with multiple health benefits (Janssen & LeBlanc 2010).

## Workplaces

### Cultivating Relational Networks that Support Prosocial Behavior

Many workplace practices involve identifying shared values, norms, and desired behaviors to promote group cohesion, cooperation, effectiveness and meaning in work (Bond et al. 2008; Chang & Lee 2007; Daniels & Daniels 2007; Houmanfar et al. 2015; Stewart et al. 2006). This process is critical to evolving an efficient organizational culture (Graham et al. 2017). It increases the groups’ focus on attaining shared long-term goals and visions, which helps improve performance (O'hora & Maglieri 2006). There is also some evidence that a focus on identifying values and related actionable behaviors is associated with increased profitability (Boyce et al. 2015), safety (McSween 2003; Moran 2015; Myers et al. 2010) and prosocial behavior (Atkins & Parker 2012). According to Burnes and Jackson (2011), around 70% of organizational change initiatives fail, and alignment of values seems to be a key part of bringing about lasting organizational change.

The work of Elinor Ostrom (Ostrom & Cox 2010; Wilson et al. 2013; Ostrom 1990) on understanding how groups can successfully manage common-pool resources over time highlights the importance of shared purpose and identity. Ostrom describes this as the first of eight core design principles characterizing a well-functioning group.

*PAX Vision* provides a structured way for workgroups to develop a shared view of which behaviors the group wants more and less of in the work environment. The process enhances group members’ commitment to the norms and goals they help in establishing (Ludwig & Geller 1997). The “see/hear/feel/do” exercise, in which group members collaborate on what they would like to see either more or less often in the workplace, is perfectly useable with adults as it is with children. Using a wall-mounted board to build the vision, with *More* sections above and *Less* sections below, is usually a good way to visualize everyone’s contributions and works as a visual reminder, especially if referred to frequently.

**Fig. 2:**
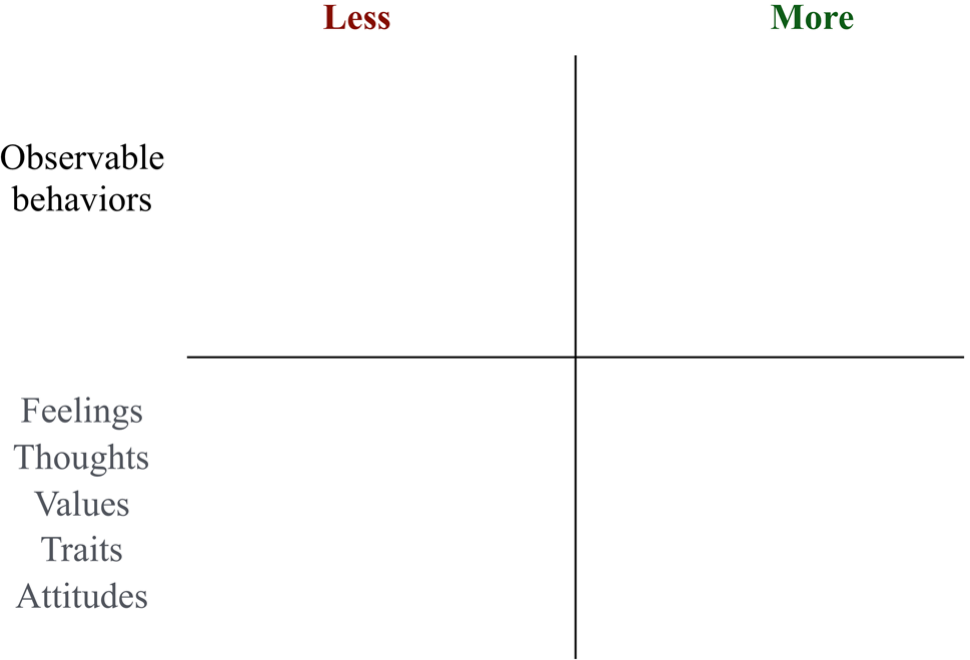
Matrix kernel

A common limitation of values work is lack of specificity. Talking in broad terms like "respect, honesty, or trust" sounds sensible and has the advantage that most people consider them desirable characteristics. But naming them does not explicitly state what people should be doing more (or less) of, which makes it open to interpretation and difficult to evaluate at the level of implementation. If we simply identify and proclaim values, it seems to have little or no impact on organizational performance (Guiso et al. 2015). We need to provide examples of clear target behaviors to observe, measure, and reinforce. Working with values to create a joint vision is a good opportunity to explicitly teach and encourage the skill of discriminating overt behaviors from the more commonly used language of traits, values, and attitudes. A simple four-field matrix (Fig. 2), inspired by combining the *PAX Vision* and the ACT Matrix (Polk et al. 2014), on a white board or on paper can help accomplish this. The person facilitating the group vision process prompts participants to give examples of specific behaviors connected to values, attitudes, and traits. For instance, if a participant says “respect” is desirable, the facilitator would write it in the bottom right quadrant, and ask the group to provide examples of behaviors (top right quadrant) promoting “respect” (for a more extensive description, see Johansson 2018). Similarly to the *PAX Vision*, this participatory process helps build and strengthen shared relational networks that can guide individuals and groups toward creating a more nurturing and productive work environment.

*PAX leader*, or *Meaningful Roles,* can be applicable in several ways. Clarifying expectations of job performance and responsibilities might seem like a prerequisite to providing a basic structure for dividing work. But in practice, employees often experience role ambiguity, which contributes to increased levels of stress, and decreased job satisfaction and performance (Patrick & Laschinger 2006; Um & Harrison 1998). Defining roles and expectations more clearly makes it easier for managers to know which behaviors to monitor/follow-up and provides frequent opportunities for (positive) feedback. Connecting a role to relevant parts of the *PAX Vision* strengthens the values process and work culture by building more dense relational networks.

### Increasing the Frequency of Reinforcement for Prosocial Behavior

*Tootle Notes,* or *praise notes,* can be given from a manager, a colleague, or anonymously, in private or in public. Increasing the frequency of positive feedback has been shown to impact work satisfaction and performance (Alvero et al. 2001; Gabelica et al. 2012; Wilk & Redmon 1998), increase connectivity in teams (Losada & Heaphy 2004), and increase efficiency if ratio of positive feedback to negative feedback is higher (about 5:1) than that of negative feedback (Losada & Heaphy 2004; Zenger & Folkman 2013).

*Granny’s Wacky Prizes* (also known as *Prize Bowl).* The use of a *prize bowl* can relate to goal setting (Agnew & Redmon 1993; Ramnerö & Törneke 2014; Squires & Wilder 2010) and function as a reinforcer for the achievement of goals or subsets of goals. If a *prize bowl* is used with rewards in the form of brief voluntary group activities that most employees perceive as fun and uplifting, this can also facilitate creativity and increase productivity (West 2015).

### Antecedents to Prosocial Behavior

*PAX Stix* can be useful in randomly selecting groups/pairs for tasks or responsibilities, or for a manager in selecting individuals to observe and reinforce (verbally or by writing) during the day. Achieving greater engagement from all group members may allow the group to tap into the potential advantages of diverse input, improving creativity, innovation, and decision-making (Horwitz & Horwitz 2007; Phillips 2003; van Knippenberg & Schippers 2007).

*PAX Quiet* can be useful at larger meetings or in training settings to save transition time. If new to the group, the kernel is most effective if introduced at the beginning of an activity, with a brief discussion or exercise planned a few minutes after introducing it, which allows for early repetition of the kernel.

### Interaction Among PAX Components

The *Predict, Monitor, and Reflect* kernel integrates relational network, antecedent, and reinforcement kernels. It is useful in many organizational settings, e.g., before and after meetings, to repeatedly establish and reinforce the components of the agreed-upon vision. The process typically takes only a few minutes at the beginning and end of a group activity. It is recommended to use *PAX Stix* to randomly select which group members speak up, to promote engagement and participation from all group members. This strengthens and helps evolve the shared relational network of behaviors that contribute to a desirable work environment.

## Criminal Justice Interventions

Current evidence indicates that many of the most common practices of the criminal justice system are harmful. These include unnecessary incarceration and unnecessarily long incarceration (Roberts 2003; Schnittker & John 2007), trauma to children whose families are in the system, and impoverishment and instability of families that result from incarceration. With respect to Juvenile Justice, evidence indicates that punitive practices are counterproductive, that therapeutic practices such as cognitive behavior therapy can reduce recidivism, but evidence-based practices are seldom employed (Lipsey et al.^2010^).

A number of the practices of the PAX system could be instrumental in improving the juvenile justice system. We illustrate this with a description of a case-challenge study involving 19 juveniles (ages 16.5 to 17.5), who had been sentenced to prison for serious, violent felony offenses. Ordinarily, 80% of paroled juvenile violent offenders returned to either juvenile or adult prisons within three months of their release. The supervising board of Pima County (Arizona) argued that they were not rehabilitating these juveniles, as much as making them into life-long criminals.

The Juvenile Facility and the County Attorney provided $90,000 to work with 19 youth to change these trajectories. Embry, an author of this paper, specifically asked for the youth deemed the worst and most likely, by the corrections officers and the teachers at the youth facility, to recidivate. The majority of the 19 youth were Hispanic and lived in barrio areas of Tucson with different gang boundaries. Two new hires were brought on, one was a male African-American youth worker and a graduating female Hispanic social worker. The youth were required to attend the program 3.5 days per week, and one juvenile parolee was required to random drug test each session.

The plan involved an intervention consisting of several evidence-based kernels, called *PAX Maps*. This intervention resulted in a reduction in the recidivism rate to 20%, compared to the regular rate of 80% recidivism for the youth remanded for serious violent offenses.

### Cultivating Relational Networks that Support Prosocial Behavior

The intervention began by helping each youth identify the goals he wanted to work on. Each engaged in multiple instances of a goal-mapping, a variation of the *PAX Vision*. It prompted the youth to identify important things they wanted to achieve, both in the short term (e.g., a couple of weeks) and in the longer term (several months to approximately a year). The *Goal/Node Map* (Collier et al. 2001; Peel & Dansereau 1998; Pitre et al. 1997) asked them to specify on the map an important goal, how and why it was important to them, what immediate measurable steps they might take toward achieving the goal, who might help them (with prompts to expand their perceived resources), what might get in the way of their efforts, and how they might meet that challenge. They revisited the process often, getting peer feedback with the group leader’s assistance.

The young men were able to elaborate further on their relational network about themselves, their behavior, and their community through activities similar to the *PAX Leader* kernel. In *PAX Leader*, students expand their prosocial behavior when they speak about their own and others’ prosocial behavior. In the juvenile justice setting, these young people became leaders, who were creating a map for their futures for things they cared about deeply. The young men earned recognition from each other, staff, and parole officers for doing “PAX.” They also learned to recognize the valued traits in others in the community, their families, and others as part of their “leadership” role.

Every participant had multiple meaningful roles for helping run the program, which included preparing meals for the whole group, creating menus, cleaning up, helping with paperwork, greeting family members or visitors, and other tasks that helped the community.

The intervention also helped to break down some verbal boundaries these young men presumably had involving gang membership. A young man’s identification with a gang can be a highly motivating relation that interferes with forming relationships with young men who may be members of other gangs while it encourages antisocial behavior that is in a hierarchical relationship with the gang name and associated symbols. To change these relational networks, each young man was randomly assigned to “crew” of five with whom they worked collaboratively.

Finally, one facet of building the prosocial relational networks of these young men involved exposing them to aspects of their community that were unfamiliar to them. The program also sought to expose the young men to many features of their community with which they had no experience. For example, they had little experience in the broader world of visiting stores, museums, political events, neighborhood events, etc. Despite their criminal pasts, few had real-world experiences beyond their perceived gang turfs. They had phobias of novel situations, which paradoxically assured they did not experience or learn about new opportunities.

### Increasing the Frequency of Reinforcement for Prosocial Behavior

Each individual learned to write positive notes (on paper or via cell phone) to other members of their crew, and other crews for prosocial behaviors. Each young man also started to write positive notes to their parole officers, counselors, educators, and even others at community events for promoting the values of PAX.

As we indicated above, the *Granny’s Wacky Prize* is a modification of the *Prize Bowl*, a particularly effective system of reinforcement (Embry & Biglan 2008). In the Pima County program, if a team member did something that was part of his goal map, he could draw from a *Prize Bowl* of small rewards. Once a day (out of four days of meeting), the facilitator would draw one name for a young man to complete a drug urinalysis. If the test was negative, the man could then draw from a “bigger” *Prize Bowl* (such as a gas or iTunes card), and the prize went to the member’s crew. In this way, the actions of one member rewarded the entire crew. Doing this created considerable peer support for not using drugs, presumably a reversal of the existing social contingencies for these young men.

Learning the actual extensive rules of parole, staying clean, and participating in potent re-entry and skill-building were gamified by having one’s crew (made up of members of rival gangs) win, too, when a member was making progress toward successful parole each day and week. The gamification increased peer cooperation and positive citizenship rather than increased deviant behavior found when such groups are aggregated (Dishion et al. 1996).

Finally, the *Prize Bowl* provided recognition for progress. It allowed chances for posting successes and opportunities to announce them and provided occasions to celebrate the success of a youth or a crew.

### Antecedents to Prosocial Behavior

*Beat the Timer* can help to focus attention on completion of many tasks, simulating future work environments or educational situations. For example, at the end of each session, we used *Beat the Timer* to motivate rapid clean-up of the room. We also used the timer to motivate rapid peer tutoring.

## Public Discussion and Government

Over the last two decades, a decreasing proportion of Americans are satisfied with the way government is functioning, moving from 68% in 2001 to 38% in 2018 (Dugan 2018). Moreover, research tracking levels of political, social, and affective polarization over time in the USA. In 2016, a Pew Research Center report showed that 45% of Republicans and 41% of Democrats viewed the other party as a “threat to the health of the nation” (Pew Research Center 2016).

The U.S. is currently flooded with what in PAX terms we might call “divisive Spleems.” We coin this neologism in the hopes of getting many more people to identify behavior that undermines our cooperation and our enhancement of well-being, without attaching anger, blame, or other negative emotional connotations to the behavior. We need to encourage an ongoing national discussion of precisely what kinds of behavior we want to see in our public discussions and governance.

### Cultivating Relational Networks that Support Prosocial Behavior

Would it be in the national interest if we promoted public discussion about what behaviors and qualities we want to *increase* in our national life and what ones we want to decrease? Decades of research on seeking ways to affect human behavior show unequivocally that it is not enough simply to identify and try to reduce antisocial behavior. We need to specify, teach, promote, model, and reinforce alternative, more desirable behavior.

We could engage people in a visioning of what we want to see, hear, do, and feel more of if the nation were moving toward becoming a better place for all Americans. The phrasing of the question is important. If you said, “America could become the best place it could be,” some would argue that it already is.

**Fig. 3:**
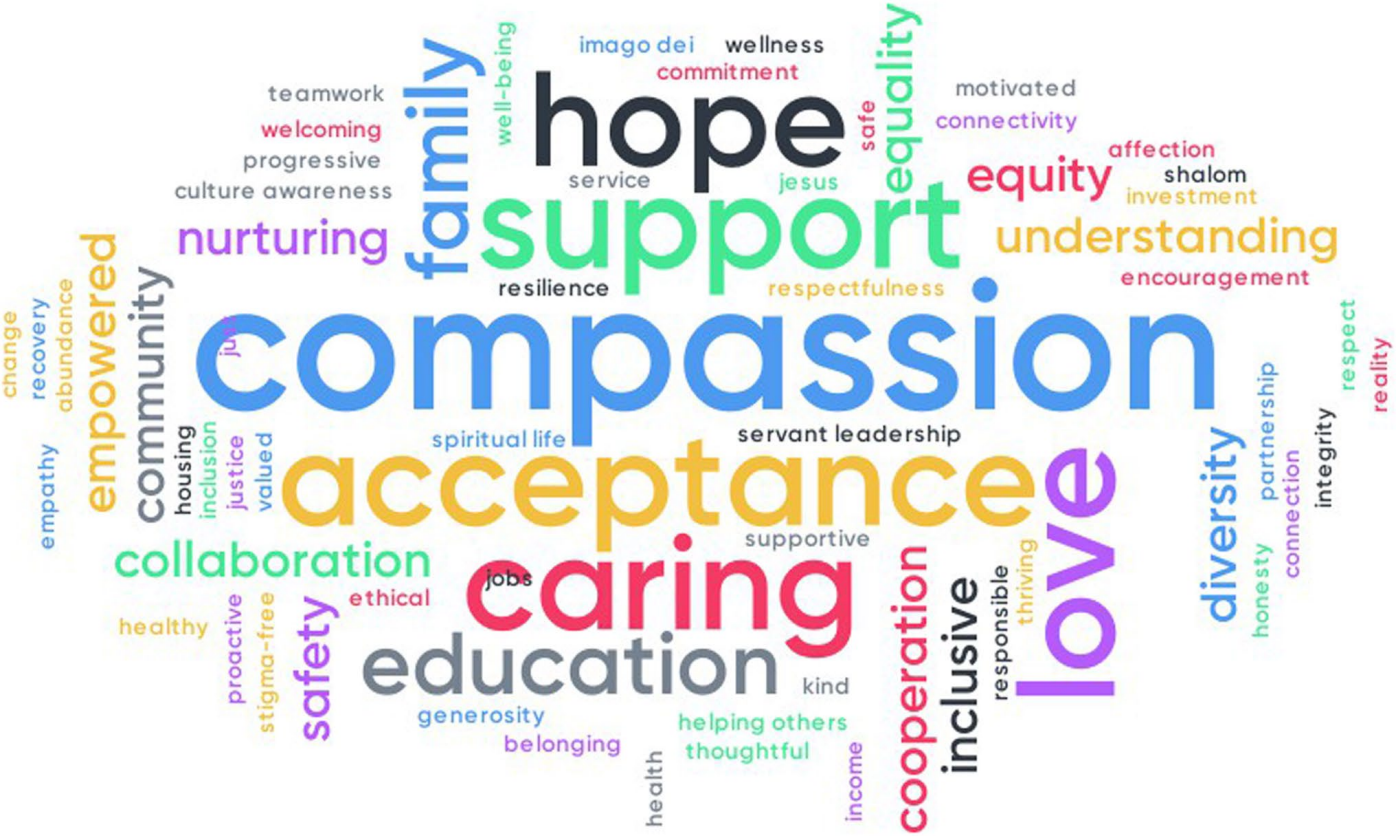
The [use color] Values identified by community leaders in a small Oregon Community. Larger words were used by more people

We have been using the practices of *PAX Vision* to get high school students and community leaders to envision what they would like more of. Figure 3 shows a word cloud for community leaders in one community in Oregon. The size of the word is a function of how many people used it. Doing word clouds for in many settings, for instance with teachers in a middle school/high school in Oregon, and with behavioral scientists and clinicians in Italy, we've found their aspirations to be very similar.

This strategy could lead neighborhoods, school boards, city councils, communities, state legislatures, and the national congress to unite around the qualities they want in their settings. Instead of focusing on policy objectives, people would focus on the qualities they want their day-to-day interactions to have. The question might be, “If [this group or body] became the most effective and satisfying one that you had ever been involved with, what would you see, hear, do, and feel more often?” With respect to neighborhoods, Gershon (2009) reports success in getting neighbors and other groups to cooperate by starting with a discussion about their aspirations for the future.

### Increasing the Frequency of Reinforcement for Prosocial Behavior

*Predict, Monitor, and Reflect* could be used in conjunction with a word cloud in an ongoing fashion to guide groups in increasing the values and behaviors they have specified in specific activities. For example, a city council might ask councilors to say what qualities they think they will see in today’s meeting. At the end of the meeting, they could do a Process Check (Glaser & Glaser 2006) in which all the councilors mention something they saw that was in keeping with the group’s goals and aspirations.

At regular intervals, members of the group would write a praise note to another member of the group. For example, a legislature might have “*Tootle Tuesday*” in which each legislator would write a note of praise to another legislator. Using the random calling procedure, the principle underlying *PAX Stix*, the group might choose at random the person who will receive the praise. Another example comes from Swedish political Twitter, where a person posted something she admired about a person whom she did not agree with politically and asked others to do the same (Azzat 2019), initiating a flow of posts with positive notes of political opponents.

We need to increase recognition for political leaders who exemplify PAX. How often have you heard a public official praise the efforts of someone who is traditionally on the opposite side of most issues? In the United States federal politics, it has been relatively rare, but there have been instances in which political leaders took steps that were conciliatory or favorable to compromise. One example is John McCain’s call for Senate Republicans and Democrats to engage in respectful discussion about their differences.

Imagine a concerted effort of a bipartisan or nonpartisan organization that did nothing more than nominating someone in our national leadership who did something conciliatory or praiseworthy from the standpoint of bringing people together. We might call them the *PAX Leader* of the day. Maybe this could contribute to changing the current nature of public debate.

## Increasing Nurturance Throughout Our Societies

The concept of evidence-based kernels lends itself well to creating and evaluating your own “recipe” of kernels, adapted to the environment you seek to improve (Embry 2011). We submit that by coordinating kernels within a relational network to promote nurturance, the collective effect of kernels can have a greater impact than their isolated effects. Most of the kernels mentioned in this paper have been subject to experimental evaluation in some settings. However, from the standpoint of variation and selection, we think the impact of any kernel should be assessed when used in any new setting. This could be as simple as assessing behavior before and after its implementation or a more elaborate multiple baseline design (Biglan et al. 2000) to assess effects in a cost-effective manner (Hawkins et al. 2007). Once the proximal benefits of a kernel or suite of kernels have been established, randomized-comparative effective trials could be employed to measure deeper or population-level outcomes. Using the kernel framework encourages openness and transparency about intervention components and can be an important step in establishing a repository of kernel efficiency research.

**Fig. 4:**
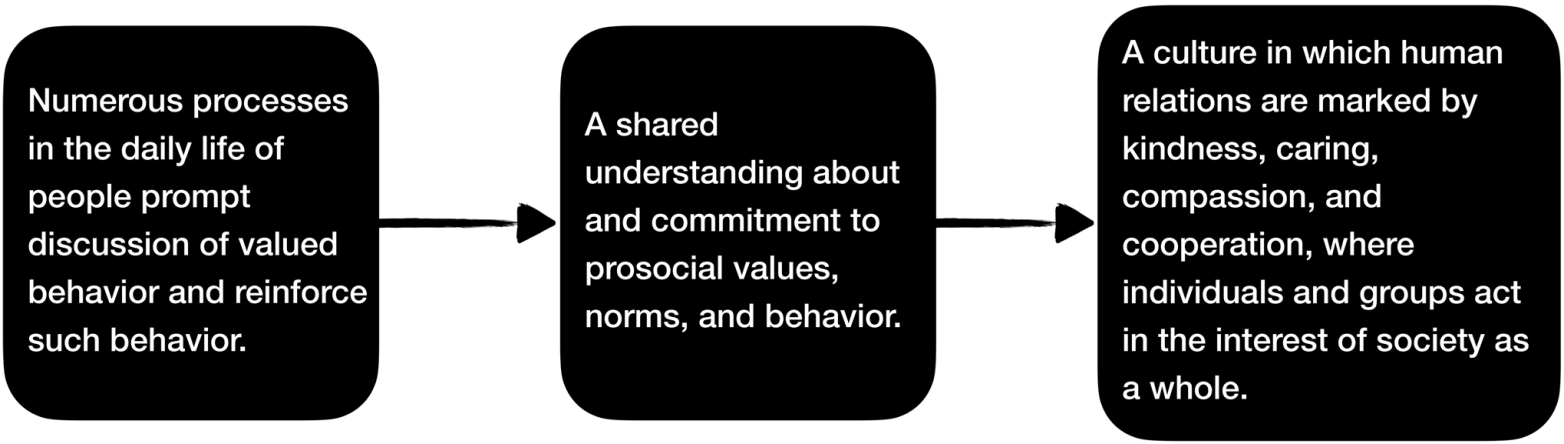
The Evolution of Nurturance in Families, Groups, Organizations, Communities, and Nations

We believe the PAX system has useful implications for how we can increase nurturance in many aspects of society. The generic principles in the processes and components of PAX GBG are relevant to families, workplaces, and group functioning generally. Figure 4 summarizes the generic features of how we can evolve a society that does a better job of nurturing people’s well-being. To the extent that we create environments that have numerous routine processes in daily life that prompt discussions of valued behavior and reinforce such behavior, we will cultivate groups that have a shared understanding about and commitment to prosocial values, norms, and behavior. And to the extent that we do this throughout society, we will evolve societies where human relations are marked by kindness, caring, compassion, and cooperation where individuals and groups act in the interest of society as a whole.
